# An Investigation of the Utility of Waist Circumference Predicting Cardiorespiratory Fitness in School Children: A Cross-Sectional Study

**DOI:** 10.3390/ijerph20010851

**Published:** 2023-01-02

**Authors:** Maria Zadarko-Domaradzka, Marek Sobolewski, Edyta Nizioł-Babiarz, Zbigniew Barabasz, Krzysztof Warchoł, Klaudia Niewczas-Czarna, Emilian Zadarko

**Affiliations:** 1 Institute of Physical Culture Sciences, Medical College of Rzeszow University, 35-959 Rzeszow, Poland; 2 Department of Quantitative Methods, Rzeszow University of Technology, 35-959 Rzeszow, Poland; 3 Department of Physical Education, Carpathian State College in Krosno, 38-400 Krosno, Poland

**Keywords:** risk factors, body composition, WC, CRF, health and well-being, prevention, H-RF

## Abstract

The early identification of modifiable risk factors and their monitoring, also within school physical education (PE) classes, are becoming indispensable in the context of public health. The aim of this study was to test whether making use of waist circumference (WC) measurements increases the possibility of predicting the results of cardiorespiratory fitness (CRF) in school-age children, as compared with body mass index (BMI) and other somatic indicators related to body fat. The cross-sectional study covered 190 children aged 10 to 15 years, participating in school PE classes. Body height (BH), body weight (BW), WC, hip circumference (HC) and percentage of body fat (BF%) were measured. BMI, waist to hip ratio (WHR) and waist to height ratio (WHtR) were calculated, and a CRF test was performed by means of a 20 m shuttle run test (20mSRT). The peak heart rate (HR_peak_) of the children was also measured. The regression model that was developed showed that WC (*R*^2^ = 47.1%), beyond BF% (*R*^2^ = 50.3%) and WHtR (*R*^2^ = 50.0%), was a useful measure of CRF, and stronger than BMI (*R*^2^ = 45.8%) or WHR (*R*^2^ = 39.2%). The risk of obtaining the CRF result classified below a good level (below the percentile range of P60-P80) was significantly higher in children with a larger WC (odds ratio (OR) for the WC change of 1 cm equals 1.14 (95% CI: 1.09–1.20; *p* < 0.001)). The simplicity of measuring WC and the possibility of using this measurement in the calculation of WHtR with reference to CRF indicate its usefulness in the prophylactic exams of school children.

## 1. Introduction

In studies concerning population health, anthropometric measurements and ratios, including body height (BH) and body weight (BW), body fat (BF), body mass index (BMI), waist to hip ratio (WHR), waist to height ratio (WHtR) and waist circumference (WC), are most commonly used as screening measures [1], as they are considered predictors of certain chronic diseases [2,3]. It has been shown that BMI and WC are essential independent factors affecting high blood pressure in children of pre-school age, in particular boys [4]. In clinical practice, the measurement of WC is not performed routinely, and the BMI alone, which is used most often, is not sufficient enough for the appropriate assessment and management of cardiometabolic risks related to obesity [5,6]. Excessive abdomen adiposis, determined on the basis of WC measurement, is a better measure of the risk of metabolic disorder occurrence in children at any age than BMI [5]. In view of the worldwide spread of the obesity problem and its health consequences, the need arises for more efficient preventive measures which would embrace mostly children and adolescents [6,7,8,9,10]. Children and adolescents with excessive visceral body fat should be carefully observed, as this type of obesity is connected with future serious health complications [5,11,12].

Among Polish 8-year-olds, 30.7% are overweight or obese; 21.5% are characterized by too large a waist circumference; and 20.8% have an increased systolic blood pressure [13]. In the years 2016–2021, Poland witnessed a significant increase in the percentage of 8-year-old children with overweight, obesity and increased values of blood pressure [7]. What is more, over the last 30 years, there has been observed a systematic decrease in the physical fitness of Polish children [14], accompanied by an increase in overweight and obesity [7,8,9]. That is why, currently in Poland, it is recommended that within PE lessons more attention should be paid to monitoring the level of physical fitness orientated at health among children and adolescents, in accordance with the concept of health-related fitness (H-RF) [15]. The elements of fitness associated with health are as follows: cardiorespiratory fitness (CRF), strength and muscular fitness and flexibility and body composition [16]. The key markers of health are CRF, considered an essential variable most strongly related to health results, and body composition components, including body fat which should be assessed in screening exams [11,12,17,18].

CRF, whose low level is strongly and independently related to all-cause mortality in adults [19,20], is an important predictor of human health. In adolescents, it is a predictor of, e.g., cardiometabolic health, premature cardiovascular disease, academic achievements and mental health [21]. A significant correlation has been found between CRF changes and the systolic blood pressure in the study of 12–18-year-olds over a period of 2 years. The adolescents with a consistently low CRF had the highest levels of systolic blood pressure [22]. CRF can be measured by laboratory tests, which are expensive and require specialized equipment, or by means of field-based tests [21], as well as by predictive equations [23]. According to a literature review, the most commonly used test for assessing CRF is the validated [24] 20mSRT, which can be conducted in big groups, e.g., in schools, and can be used to monitor the health of the population [19,25,26], though not all of the researchers support this view [27]. Lang et al. (2018) recommend this test as a holistic health marker which can help to identify children and adolescents at risk of poor health [28]. The results of cross-sectional studies imply a correlation between body fat and the CRF level. Body fat negatively influences CRF levels among children and adolescents with normal BMI [29]. Hence, the early identification of modifiable risk factors and monitoring them among the population of children by means of simple measures is becoming a modern necessity in the scope of public health [30,31]. In Polish schools, in accordance with the current core curriculum of general education in PE, within PE lessons, physical fitness, BH and BW are assessed, which only allow for calculating the BMI [32]. There are studies, however, which point out that determining the BMI alone is insufficient due to the lack of reference of this index to body fat [33].

The aim of this study was to test whether making use of the WC measurement increases the possibility of predicting CRF results in school children, as compared to the BMI and other somatic indicators related to body fat.

## 2. Materials and Methods

### 2.1. Participants and Study Procedures

The study covered school children within the age group 10–15 years, without any medical contraindications to participating in school PE lessons. The sampling was purposive, and the tests were performed in a school with which the Physical Education Department (WWF) of Rzeszow University (UR) had entered a cooperation agreement. The tests were performed in the school’s venue, in the morning. Prior to the tests, written consent of the school authorities, of the parents or legal guardians and of the children was obtained. The children whose parents did not consent to the tests and those who did have the consent yet were absent on the days of the tests were excluded from the study. The participants of the study were informed about the purpose and the procedure of the trials and measurements. Within the study, anthropometric measurements were taken, and a cardiorespiratory fitness test was performed. Ultimately, 190 school children (41.6% of whom were boys and 58.4% were girls) participated in the tests. The age of the tested children was determined based on the information provided by the parents. The study was conducted in 2017 with the approval of the Rzeszow University bioethics committee (No. 1/06/2014). 

### 2.2. Anthropometric Measurements

In accordance with the measuring protocol adopted, body height was measured by means of the Seca 213 stadiometer (Seca GmbH & Co. KG., Hamburg, Germany) with accuracy to 0.1 cm. Body weight and BF% were determined through an electrical bioimpedance analysis with the use of the Tanita TBF 300 (Tanita Corporation, Tokyo, Japan): a body composition analyzer. These measurements also provided information on the BMI expressed as BW (kg)/BH squared (m). The BMI was classified according to WHO criteria [34]. Waist circumference and hip circumference (HC) were measured by means of the Gulick anthropometric tape (BASELINE) with accuracy to 0.1 cm. The WC measurement was performed midway between the bottom edge of the lower rib and the upper iliac crest. Hip circumference was measured at the maximum circumference over the buttocks. Circumference was measured in a standing position with arms resting at the side of the body and weight evenly distributed between the feet. The children were barefoot and lightly dressed. All measurements were performed twice and the arithmetic mean of the two measurements constituted the final value [11,35]. The measurements were performed in circumstances providing privacy and respecting the dignity of the tested children: they were measured individually, with boys and girls separately. The WHR was calculated by dividing the WC (cm) by the HC (cm). The WHtR was calculated as WC (cm)/BH (cm).

### 2.3. Cardiorespiratory Fitness Measurement

The cardiorespiratory fitness was assessed on the basis of the 20 m shuttle run test [36]. The test consisted of performing a shuttle run (‘back and forth’) over the distance of 20 m. The distance had been established with a measuring tape and marked with a line and cones. The speed of the run was regulated by means of audio signals which occurred more and more frequently.

The starting speed of the run was 8.5 km/h, and it was increased by 0.5 km/h with each stage. The tested person was supposed to move in such a way as to be at one of the two ends of the 20 m line at the moment of hearing the signal. In the case of school-aged children, it is recommended that they run with a leader—a pacemaker—who helps them to keep up with the audio signals. This recommendation was followed in our study. The aim of the trial was for the participant to maintain the set rhythm for as long as possible. The result of the test was the number of fully completed 20 m laps. The trial was terminated the moment the participant failed to maintain the imposed rhythm twice in a row or declared himself/herself unable to continue. 

All the participants were provided with identical conditions and the test was conducted in the school sports hall. At the end of the 20 m shuttle test, the peak heart rate of the children (HR_peak_; beats per minute) was established by means of the Polar Team2 system (Polar Electro Oy, Kempele, Finland).

### 2.4. Data and Statistical Analysis

The number of laps completed by the participants referred to the centile norms as suggested by Tomkinson [37]. In order to facilitate the interpretation of the results, the numbers of laps run were classified into a 7-stage adjective scale, with respect to centile norms specific to sex and age. The conversion used was <P5 = very poor, P5–P20 = poor, P20–P40 = fair, P40–P60 = average, P60–P80 = good, P80–P95 = very good and >P95 = excellent, where P indicates percentiles.

For the presentation of the findings, the tested children were divided into 3 age groups: 10–11 years, 12–13 years and 14–15 years, and in the statistical analysis (correlations, regression models) age was treated as a quantitative variable. 

In the descriptive statistics of the variables, arithmetic means (M) were used, together with standard deviations (SD) as well as minimum (Min) and maximum (Max) values. In order to determine correlations between the somatic indicators and the results of the cardiorespiratory fitness test, a regression analysis was applied. Five models were created, in which each of the somatic indicators (BMI, BF%, WHR, WHtR and WC) was consecutively introduced as an independent variable. The models also considered sex and age. Comparisons of the predictive value of particular somatic indicators were made on the basis of the coefficient of determination (*R*^2^). For example, for one-way comparisons of CRF centile classifications among girls and boys, and with respect to age, the Mann–Whitney test was used, alongside Spearman’s rank correlation coefficient. A logistic regression model was also created, by means of which the influence of WC on the probability of the occurrence of the CRF value classified below the good level (P60–P80 = good) was assessed. The results of the logistic regression analysis were presented by means of an odds ratio (OR). In the interpretation of the results, the values of *p* ≤ 0.05 were considered as statistically significant. All analyses were performed in the Statistica 13.3 software (TIBCO Software Inc., Palo Alto, CA, USA).

## 3. Results

### 3.1. Test Group Characteristics

The analysis concerns a group of 190 school children aged 10 to 15 years. The group consisted of 111 girls and 79 boys. The age distribution in both groups was similar: the mean age of the girls was 12.5 ± 1.8 years, and 12.4 ± 2.0 in the case of the boys. According to the BMI classification ranges [34], 24% of the tested group was overweight and 18% was obese. Among the girls, 32% were overweight and 12% were obese; among the boys, they were 13% and 27%, respectively. The characteristics of the tested cohort with respect to the somatic indicators and the CRF test results (number of laps) are presented in Table 1. 

### 3.2. Classification of the Cardiorespiratory Fitness for the Groups of Girls and Boys

By means of the regression analysis, it was shown that the results of the 20 m shuttle run test (20mSRT) viewed as the number of laps covered were significantly higher among the boys (*p* < 0.001). They were also significantly varied with respect to the age (*p* < 0.001). Based on the value of regression coefficients it can be noted that, on average, the number of laps covered by the boys was higher than that of the girls by 17.4. Furthermore, with each year of age, the results of the school children increased by 4.4 laps (Table 2).

Next, considering sex and age, the numbers of laps run were classified into the 7-point adjective scale and presented for the whole cohort and separately for the girls and for the boys (Table 3). On the basis of the Mann–Whitney test result (*p* = 0.643), it can be said that with reference to the centile norms adopted from Tomkinson, the CRF of the boys and the girls was at a similar level. Most results obtained by the school children were at least at an average level. It was also verified how correlated the CRF classification was to age: for that purpose, the values of Spearman’s rank correlation coefficient were calculated. A statistically significant, yet weak, correlation between the fitness level and the age existed in the group of girls (*r*_S_ = 0.28).

### 3.3. Correlations between Somatic Indicators Measured in the School Children and Cardiorespiratory Fitness

The above-presented analyses suggest that the number of laps is influenced by the sex and the age of the school children; hence, five separate regression models were created in which the number of laps was a dependent variable while sex, age and, in turn, each of the considered somatic indicators BMI, BF%, WC, WHR and WHtR were the independent factors.

Table 4 presents the values of the coefficient of determination (*R*^2^) for each of the models (additionally, the F statistic value and the *p* value for the significance of the whole model). All of the models were statistically significant, and their predictive value was very similar: the coefficient of determination ranged between 39.2 and 50.3%.

The regression model that was developed showed that WC (*R*^2^ = 47.1%), alongside BF% (*R*^2^ = 50.3%) and WHtR (*R*^2^ = 50.0%), was a useful measure of CRF, and stronger than BMI (*R*^2^ = 45.8%) or WHR (*R*^2^ = 39.2%).

**Figure 1:**
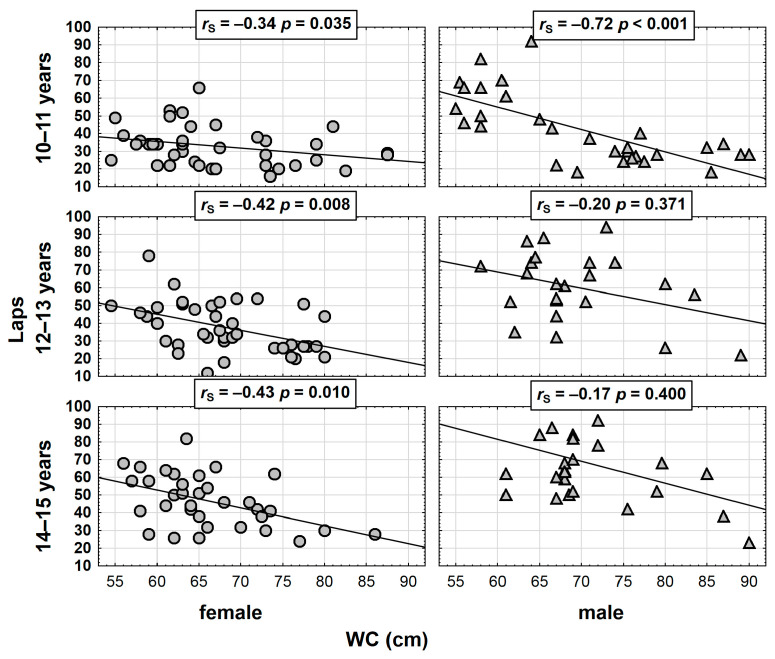
Distribution of CRF test results (number of laps) with reference to the WC for particular age groups among the girls and the boys.

The correlation between WC and the number of the 20 m laps run is presented by means of the scatter graph, with regard to sex and age (Figure 1).

Table 5 presents the coefficients of the regression model by means of which one could perform the preliminary estimation of the number of laps on the basis of the information on the sex, age and waist circumference of the child. With the variable WC, this coefficient equalled −0.9, which means that each centimeter of waist circumference translated into the decrease in the number of laps by this value. Boys of the same age and with the same WC had, on average, the number of laps higher by approx. 20, and with each year of age, the expected increase in the number of laps was approx. 4.3.

Among the tested school children, a group was singled out in which CRF results (number of laps) were classified below the good level (below P60–P80). Next, a logistic regression analysis was applied to estimate the odds ratio (OR) for the occurrence of CRF below the good level with the WC change of 1 cm. The OR value amounted to 1.14 (95% CI: 1.09–1.20; *p* < 0.001). That means that 1 cm of waist circumference translated, on average, into the increase in the risk of the occurrence of CRF below the good level by 14%. In the case of a difference in the waist circumference of 5 cm, the increase in the risk of being in the CRF group at the level below the good one was almost twofold (1.14^5^ = 1.92), and with 10 cm of the difference, this increase was nearly fourfold (1.14^10^ = 3.70).

## 4. Discussion

Research aims at finding more than one simple-to-measure indicator which, when combined with BMI, allows for a better estimation of a health risk measure [26,33,38,39,40]. It has been noted that over the last years, abdominal adiposis in infants, children and adolescents has increased more than general adiposis, which indicates that the frequency of obesity’s occurrence may be underestimated, if it is based exclusively on BMI [5]. The possibility to precisely assess body fat, especially the central one, requires specialized diagnostic equipment; hence, in practice, in order to determine BF distribution, WC as well as WHtR and WHR are used [11]. In the recommendations of the International Atherosclerosis Society (IAS) and International Chair on Cardiometabolic Risk (ICCR) Working Group on Visceral Obesity it can be read “that measurements of waist circumference and BMI should become a standard part of clinical encounters (that is, an accepted ‘vital sign’)” [6].

It is emphasized that the measurement of WC is the simplest, and at the same time, the most effective measure of truncal obesity in children and adolescents in comparison with WHR [41]. In our studies, WHR turned out to be the least sensitive predictor of CRF (*R*^2^ = 39.2%).

It is more and more often suggested that it is WHtR that can be a more effective indicator of ‘early health risks’ related to central obesity [42]. WHtR is also indicated as a much better predictor of cardiovascular diseases than BMI and WC [43,44]. In our studies, WHtR also turned out to be a stronger predictor for the CRF result in comparison with BMI and WC. Research suggests that the measurement of BF through BIA and manual methods for measuring WC are interchangeable [45]. The results of the studies presented here suggest that the predictive value for the CRF result with reference to the measures WC (*R*^2^ = 47.1%) and WHtR (*R*^2^ = 50.3%) was close to the predictive value of the BF% (*R*^2^ = 50.3%) estimated by means of bioimpedance. In addition, in other studies comprising children, significant correlates of BF and WC were noticed [46].

The condition of CRF in the population of children and adolescents may help to predict future diseases, including heart diseases, cancers, chronic respiratory diseases or diabetes [47]. A review of studies suggests that better results in the 20 m shuttle run test (20mSRT) are correlated with various positive health indicators, from lower levels of obesity to positive cardiometabolic biomarker profiles and physical self-assessment [28]. In the study presented here, the highest number of people in total (23.2%) was classified into the average CRF range (P40–P60). The total percentage of the school children below the good level of CRF (P60–P80) amounted to 40%. The risk of obtaining the CRF result classified below the good level (below the percentile range of P60–P80) was significantly higher in the school children with a larger WC. One centimeter of waist circumference translated, on average, into the increase in the risk of CRF below the good level by 14%. In the case of a 5 cm difference in the waist circumference, the increase in the risk of being in the CRF group at the level below the good one was almost twofold, and with the 10 cm difference, it was as high as almost fourfold. The Polish Institute of Mother and Child, in its latest report (2022) on the health of children, recommends considering the introduction of WC measurement and calculating WHtR into the current national screening test for detecting disorders in the physical development of the student [7]. CRF should be an important aim already at a young age, preventing general and abdominal obesity in the future [48].

As other authors point out, school may play an important role in identifying children with a lower level of physical fitness [16,30]. The assessment of physical fitness, in accordance with the H-RF concept, as well as the measurements of body height and body mass, together with their interpretation (allowing only for calculating general obesity indicator, BMI), are obligatory in Poland for all children above 10 years of age, within PE lessons [32]. There are, however, no health recommendations concerning body mass, e.g., BF or WC measurement. The results of our analyses showed that BF% and WHtR are stronger predictors of the CRF result and their obtainment requires the use of specialized measuring equipment or numerical computations. That is why the measurement of WC, due to its simplicity as well as the possibility of using the results for calculating WHtR can be commonly performed within school PE lessons and included and recommended in the core curriculum for this subject. According to McCarthy (2006), the information that can be obtained from the waist measurement of children, as well as adults, together with the recent changes in the distribution of body fat should constitute an impulse to perform it routinely [5]. It is emphasized that early interventions and supporting prophylactics (e.g., in schools) aimed at promoting CRF of adolescents can help children in attaining and maintaining better health later in life [49].

Rollo et al. (2022) note that currently there is too broad a range of health-related cut-points with reference to CRF among adolescents to indicate universal thresholds specific to age and sex [50]. Similarly to other countries [30,40,51], in the population of Polish children there is undoubtedly a need for broader studies with reference to current cut-points for cardiorespiratory fitness associated with overweight/obesity in children, serving the purpose of identifying children and adolescents at risk of bad health. Within studies of that type, it is worth considering an opinion about applying stricter and more scientific assessments of CRF [27].

In our opinion, a strong point of this study consists of the fact that the participants performed the 20 m shuttle test with full commitment, which can be proved by the high mean values of HR_peak_ at the end of the test, which implies that not only the school children who are more physically active were motivated to do the test. What is more, to the best of our knowledge, little research is available covering the population of Polish children, which would take into consideration in one study (such as in our research) the results of CRF with reference to several, commonly used, simple screening measures for the health of the population. For example, in one big Polish population study on children and adolescents, the 20 m shuttle run test was performed within the Eurofit test, but only BH and BW were measured and BMI calculated [52]. On the other hand, in recently published studies covering Polish children with overweight and obesity, the results of CRF measured with a laboratory test referred to BF% and BMI, but not to the other three indicators used in our study [53].

The limitation of this study is the relatively small population of children from one school, which still allowed for the adopted CRF classification to be represented by both sexes in all the adopted centile CRF categories except for the ‘very poor’ one. However, due to its cross-sectional character, this study cannot determine the causality of the relation between CRF and the anthropometric indicators, hence the need for further longitudinal studies covering a bigger sample. Despite its limitations, the value of our study was to provide relevant data on the health of school-aged children.

## 5. Conclusions

It has been shown that waist circumference, alongside BF% and WHtR, is a useful CRF predictor. Additionally, it has been demonstrated that somatic indices related to BF%, except for WHR, were stronger CRF predictors than BMI, which confirm the claim that BMI should not be the only index used. WC measurement allows the additional usage of its result for calculating WHtR, whose predictive value with respect to CRF was, in our study, at the level of BF% measured by means of BIA, which indicates its usefulness in the prophylactic exams of school children.

## Figures and Tables

**Table 1:** Test group characteristics.

Measure	Sex	Age (Years)											
		10–11 (N = 68)				12–13 (N = 62)				14–15 (N = 60)			
		M	SD	Min	Max	M	SD	Min	Max	M	SD	Min	Max
BH (cm)	F	152.5	8.0	134.0	165.0	160.5	6.4	149.0	173.5	162.3	5.6	149.0	172.5
	M	148.2	7.6	131.0	168.0	163.2	10.1	148.3	180.0	172.9	7.6	160.4	190.4
BW (kg)	F	47.9	11.1	31.4	72.9	54.2	8.3	39.4	70.5	53.9	11.2	39.6	93.6
	M	46.2	12.1	24.2	67.9	53.1	14.0	34.2	84.9	60.3	11.7	41.2	88.2
HC (cm)	F	86.0	8.6	72.5	100.5	91.6	6.2	80.0	103.5	92.2	9.8	79.5	124.5
	M	83.2	10.9	65.0	102.5	86.0	9.6	71.5	108.0	90.4	7.6	79.0	104.0
BMI	F	20.5	3.9	15.0	30.3	21.0	3.0	16.0	27.2	20.5	4.1	15.5	32.0
	M	20.9	4.7	14.1	29.6	19.7	3.7	15.2	29.0	20.1	3.5	14.6	27.6
BF%	F	23.6	7.5	8.0	37.3	26.2	6.1	9.0	40.2	21.8	7.5	7.1	37.1
	M	19.0	9.8	5.1	35.4	12.0	6.6	3.6	29.1	11.0	7.2	1.4	29.1
WHR	F	0.78	0.06	0.68	0.96	0.74	0.05	0.63	0.84	0.72	0.03	0.66	0.78
	M	0.84	0.05	0.72	0.94	0.81	0.05	0.74	0.93	0.79	0.05	0.72	0.90
WHtR	F	0.44	0.06	0.37	0.59	0.42	0.04	0.35	0.51	0.41	0.05	0.34	0.50
	M	0.47	0.07	0.38	0.60	0.43	0.04	0.37	0.58	0.41	0.04	0.36	0.55
WC (cm)	F	67.3	8.8	54.5	87.5	68.1	6.9	54.5	80.0	66.1	6.9	56.0	86.0
	M	70.1	11.0	55.0	90.0	69.5	7.6	58.0	89.0	71.2	7.3	61.0	90.0
Laps	F	32.8	11.4	16.0	66.0	37.8	14.0	12.0	78.0	46.7	14.8	24.0	82.0
	M	42.3	19.7	18.0	92.0	60.2	19.4	22.0	94.0	67.7	21.7	23.0	112.0
HR_peak_ (bpm)	F	197.4	6.9	180.0	213.0	195.7	9.5	176.0	213.0	194.5	9.2	177.0	216.0
	M	200.6	5.6	190.0	210.0	200.4	8.3	185.0	219.0	198.0	7.0	183.0	212.0

F—female, M—male, BH—body height, BW—body weight, HC—hip circumference, BMI—body mass index, BF%—body fat percentage, WHR—waist to hip ratio, WHtR—waist to height ratio, WC—waist circumference, Laps—laps of the 20 m shuttle test, HR_peak_—heart rate peak, M—mean, SD—standard deviation, Min—minimum values, Max—maximum values.

**Table 2:** Regression analysis results: sex and age vs. fitness test results (number of laps).

Independent Variables	Laps *R*^2^ = 34.7% *F* = 49.6 *p* < 0.001	
	*B* (95% CI)	*p*
Sex (male vs. female)	17.4 (12.6; 22.2)	<0.001
Age (years)	4.4 (3.1; 5.6)	<0.001

*R*^2^—coefficient of determination; *F*—test statistic and *p* value for significance of whole model; *B*—regression coefficient with 95% CI; *p* value for significance of each regression coefficient; Laps—laps of the 20 m shuttle test.

**Table 3:** Classification of cardiorespiratory fitness with regard to age and sex of the school children as well as to centile norms.

Classification CRF	Sex (*p* = 0.643)		Total (N = 190) N (%)
	Female (N = 111) N (%)	Male (N = 79) N (%)	
very poor (<P5)	0 (0.0)	0 (0.0)	0 (0.0)
poor (P5–P20)	2 (1.8)	4 (5.1)	6 (3.2)
fair (P20–P40)	16 (14.4)	10 (12.7)	26 (13.7)
average (P40–P60)	30 (27.0)	14 (17.7)	44 (23.2)
good (P60–P80)	19 (17.1)	18 (22.8)	37 (19.5)
very good (P80–P95)	25 (22.5)	18 (22.8)	43 (22.6)
excellent (>P95)	19 (17.1)	15 (19.0)	34 (17.9)
*r*_s_ (*p*)	0.28 (0.003)	0.17 (0.126)	0.23 (0.001)

*p* value calculated using Mann–Whitney test, P—percentiles, *r*_s_ (*p*)—Spearman coefficient of correlation with *p* value.

**Table 4:** Predictive values of the CRF test (number of laps) with reference to particular somatic indicators: the results of regression analysis.

Model	Independent Factors	Statistics of Regression Models		
		R^2^	F	p
1	Age, sex, BMI	45.8%	54.2	<0.001
2	Age, sex, BF%	50.3%	64.9	<0.001
3	Age, sex, WC	47.1%	57.2	<0.001
4	Age, sex, WHR	39.2%	41.6	<0.001
5	Age, sex, WHtR	50.0%	64.1	<0.001

*R*^2^—coefficient of determination, *F, p*—test statistic and *p* value for significance of whole model, BMI—body mass index, BF%—body fat percentage, WC—waist circumference, WHR—waist to hip ratio, WHtR—waist to height ratio.

**Table 5:** Results of regression analysis: sex and age vs. fitness test results (number of laps).

Independent Variables	Laps *R*^2^ = 47.1% *F* = 57.2 *p* < 0.001	
	*B* (95% CI)	*p*
Intercept	46.1 (23.5; 68.8)	<0.001
Sex (male vs. female)	20.1 (15.8; 24.5)	<0.001
Age (years)	4.3 (3.2; 5.4)	<0.001
WC (cm)	–0.9 (–1.2; –0.6)	<0.001

*R*^2^—coefficient of determination; *F*—test statistic and *p* value for significance of whole model; *B*—regression coefficient with 95% CI, *p* value for significance of each regression coefficient; Laps—laps of the 20 m shuttle test; WC—waist circumference.

## Data Availability

The data of the present experimental study can be available from the corresponding author via reasonable request.
